# The complete chloroplast genome sequence of *Beckmannia syzigachne*

**DOI:** 10.1080/23802359.2020.1867013

**Published:** 2021-02-08

**Authors:** Yuan Zheng, Maniya Luo, Yunqin Li, Yi Wang

**Affiliations:** a College of Forestry, Southwest Forestry University, Kunming, Yunnan, People's Republic of China; b Laboratory of Forest Plant Cultivation and Utilization, Yunnan Academy of Forestry & Grassland Science, Kunming, Yunnan, People's Republic of China

**Keywords:** *Beckmannia syzigachne*, chloroplast, Illumina sequencing, phylogenetic analysis

## Abstract

The first complete chloroplast genome (cpDNA) sequence of *Beckmannia syzigachne* was determined from Illumina HiSeq pair-end sequencing data in this study. The cpDNA is 136,181 bp in length, contains a large single-copy region (LSC) of 80,345 bp and a small single-copy region (SSC) of 12,810 bp, which were separated by a pair of inverted repeats (IR) regions of 21,513 bp. The genome contains 132 genes, including 85 protein-coding genes, 8 ribosomal RNA genes, and 39 transfer RNA genes. Further phylogenomic analysis showed that *B. syzigachne* clustered in a unique clade in the Pooideae subfamily.

*Beckmannia syzigachne* is the species of the genus *Beckmannia* within the family Poaceae. It is born in wetland, ditch and shallow water below 3700 m above sea level. It spreads all over the world (Cui et al. 2016). It is one of the main weeds in tin wheat and rape fields. In recent years, *B. syzigachne* has developed into a malignant weed in wheat and rape fields (Du et al. 2016; Pan et al. 2016). However, there has been no genomic studies on *B. syzigachne.*

Herein, we reported and characterized the complete *B. syzigachne* plastid genome. The GenBank accession number is MT653696. One *B. syzigachne* individual (specimen number: 2020023) was collected from Kunming, Yunnan Province of China (25°13′23″N, 102°72′12″E). The specimen is stored at Yunnan Academy of Forestry Herbarium, Kunming, China, and the accession number is jlq187. DNA was extracted from its fresh leaves using DNA Plantzol Reagent (Invitrogen, Carlsbad, CA, USA).

Paired-end reads were sequenced by using Illumina HiSeq system (Illumina, San Diego, CA). In total, about 23.17 million high-quality clean reads were generated with adaptors trimmed. Aligning, assembly, and annotation were conducted by CLC de novo assembler (CLC Bio, Aarhus, Denmark), BLAST, GeSeq (Tillich et al. 2017), and GENEIOUS v 11.0.5 (Biomatters Ltd, Auckland, New Zealand). To confirm the phylogenetic position of *B. syzigachne*, other 18 species of *Pooideae* subfamily from NCBI were aligned using MAFFT v.7 (Katoh and Standley 2013). The Auto algorithm in the MAFFT alignment software was used to align the 21 complete genome sequences and the G-INS-i algorithm was used to align the partial complex sequences. The maximum likelihood (ML) bootstrap analysis was conducted using RAxML (Stamatakis 2006); bootstrap probability values were calculated from 1000 replicates. *Dendrocalamus sinicus* (MK962316) and *D. latiflorus* (FJ970916) served as the out-group.

The complete *B. syzigachne* plastid genome is a circular DNA molecule with the length of 136,181 bp, contains a large single-copy region (LSC) of 80,345 bp and a small single-copy region (SSC) of 12,810 bp, which were separated by a pair of inverted repeats (IR) regions of 21,513 bp. The overall GC content of the whole genome is 38.4%, and the corresponding values of the LSC, SSC, and IR regions are 36.4%, 32.6%, and 43.9%, respectively. The plastid genome contained 132 genes, including 85 protein-coding genes, 8 ribosomal RNA genes, and 39 transfer RNA genes. Phylogenetic analysis showed that *B. syzigachne* clustered in a unique clade in the *Pooideae* subfamily (Figure 1). The determination of the complete plastid genome sequences provided new molecular data to illuminate the *Pooideae* subfamily evolution.

**Figure 1.**
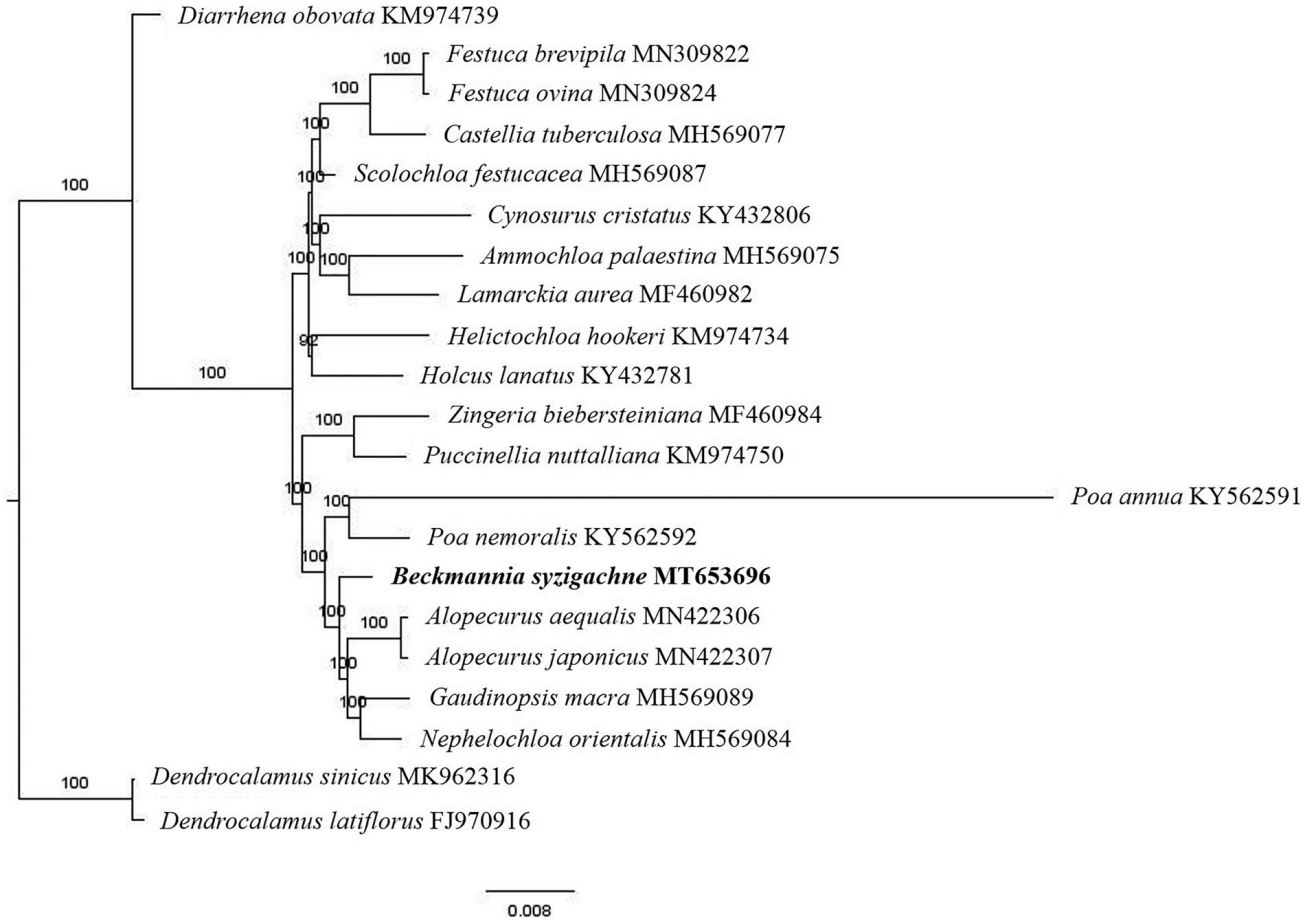
The maximum-likelihood tree based on the nineteen chloroplast genomes of subfamily *Pooideae*. The bootstrap value based on 1000 replicates is shown on each node.

## Data Availability

The data that support the findings of this study are openly available in NCBI GenBank database at (https://www.ncbi.nlm.nih.gov) with the accession number is MT653696, the associated BioProject, SRA, and Bio-Sample numbers of the raw sequence data are PRJNA663416, SAMN16132010 and SAMN16132011, respectively, which permits unrestricted use, distribution, and reproduction in any medium, provided the original work is properly cited.
